# Genome-wide association study of nutrient composition in meat from three two-way crossbred pig populations using whole-genome resequencing

**DOI:** 10.3389/fvets.2026.1758076

**Published:** 2026-01-29

**Authors:** Jie Tang, Yan Liang, Rui An, Gan Luo, Xuan Tao, Pengliang Liu, Yiren Gu

**Affiliations:** 1 Key Laboratory of Qinghai-Tibetan Plateau Animal Genetic Resource Reservation and Utilization, Ministry of Education, Southwest Minzu University, Chengdu, China; 2 Animal Genetic Breeding and Reproduction Key Laboratory of Sichuan Province, Sichuan Animal Science Academy, Chengdu, China

**Keywords:** amino acid, crossbred pig population, fatty acid, genome-wide association study, whole-genome resequencing

## Abstract

Pork is a major source of animal protein for humans, and as living standards have improved, consumer demand has shifted from quantity to quality. Amino acid and fatty acid compositions determine the nutritional value and flavor of pork. However, the genetic mechanisms underlying variation in these parameters have not been fully elucidated. In this study, we quantified 17 amino acids and 42 fatty acids in the *longissimus dorsi* muscle from three crossbred pig populations, namely Yorkshire × Tibetan (YT), Yorkshire × Neijiang (YN), and Duroc × Tibetan (DT). YT and YN pigs exhibited higher amino acid concentrations, while DT pigs showed elevated fatty acid levels. Subsequently, whole-genome resequencing of 73 pigs identified 24,125,658 high-quality SNPs, among which 146 were significantly associated with fatty acid traits, leading to the identification of 19 candidate genes linked to palmitic acid (i.e., *GALNT2, RET, RHOU, PHYHIPL, FAM13C, BICC1*, and *TAF5L*), oleic acid (i.e., *ABCB10, LRP1B, ZNF37A, RHOBTB1, HNRNPF, TMEM26, URB2, FXYD4, PGBD5, LOC110256649*, and *LOC110256821*), and total fatty acids (i.e., *UBE2E2*). Functional annotation revealed that these candidate genes participate primarily in pathways related to lipid metabolism, glucose homeostasis, and energy balance. The identified SNPs and candidate genes provide valuable insights into the genetic architecture of the fatty acid composition in pork and may serve as molecular targets for improving meat quality through breeding.

## 1 Introduction

In response to increasing consumer demand for high-quality pork, the main goal of pig breeding programs has transitioned from improving growth rate to meat quality (1, 2). The amino acid and fatty acid compositions are closely related to quality characteristics, including the nutritional value and flavor of pork. Typically, essential amino acids (e.g., lysine and tryptophan) participate in protein synthesis and sustain vital physiological processes, while other amino acids (e.g., glutamic acid and alanine) are critical for shaping sensory properties, including flavor and taste. From a nutritional aspect, monounsaturated fatty acids (e.g., C18:1n9c) and polyunsaturated fatty acids (e.g., C18:2n6c) are beneficial, as they improve lipid metabolism and reduce inflammatory responses (3, 4). In contrast, excessive intake of saturated fatty acids (e.g., C14:0 and C16:0) may increase the risk of cardiovascular disease and type 2 diabetes in humans (5–7). With respect to meat quality, the content of saturated fatty acids is associated with the melting point and firmness of meat fat, whereas monounsaturated fatty acids (e.g., C18:1n9c) can enhance meat color and flavor (8, 9).

Recent genetic studies, including genome-wide association studies (GWAS), have revealed a large number of genetic loci and candidate genes associated with porcine meat quality traits, providing insights into the genetic basis of meat quality (10, 11). However, research in this field has several limitations. For example, studies have focused on common meat quality traits, such as pH, meat color, water-holding capacity, tenderness, and marbling, with relatively few precise analyses of fatty acid and amino acid contents (12, 13). Additionally, few studies have evaluated crossbred pigs of Western and Chinese indigenous breeds, which produce progeny that combine rapid growth and good meat quality (14–18). Many studies have used pig populations with relatively low genetic diversity, and the use of genotyping arrays may miss key genetic variants (19, 20).

Meat quality traits are closely regulated by amino acid and fatty acid profiles and are key targets for swine genetic improvement. In this study, we determined the amino acid and fatty acid contents in the *longissimus dorsi* muscle from three crossbred pig populations. Based on whole-genome resequencing data for 73 pigs, we performed a GWAS to identify single nucleotide polymorphisms (SNPs) and candidate genes associated with amino acid and fatty acid contents. These analyses provide insights into the effects of amino acids and fatty acids on meat quality traits across crossbreeding combinations and the molecular mechanisms regulating the fatty acid content in crossbred pigs.

## 2 Materials and methods

### 2.1 Animals and sample preparation

We used 73 healthy F_1_ crossbred pigs from three populations, including 33 from Yorkshire boar × Tibetan sow (YT; 15 sows and 18 barrows), 20 from Yorkshire boar × Neijiang sow (YN; 10 sows and 10 barrows), and 20 from Duroc boar × Tibetan sow (DT; 18 sows and two barrows). During the experimental rearing period, all pigs were housed in the same experimental pig farm at 10–11 pigs per pen, with a consistent stocking density per pen for the three populations. All pigs were fed twice daily with the same basal diet (corn-soybean-based diet containing 16% crude protein, 13.0 MJ/kg digestible energy, and 0.78% lysine) and had access to water *ad libitum*. Before slaughter, all pigs were fasted for 24 h with free access to water. The pigs were centrally slaughtered at the same abattoir on a single day, at an average age of 180 days, with an average body weight of 96.10 ± 0.91 kg (mean ± SEM). The slaughter procedures followed the specifications described in the Operating Procedures of Livestock and Poultry Slaughtering-Pig (GB/T 17236–2019). Specifically, we employed electrical stunning for pig slaughter, ensured the pig remained unconscious with a heartbeat, and immediately performed sticking and exsanguination within 30 s after stunning. For each pig, ear tissue (approximately 0.5 g) was collected, preserved in 75% ethanol, and stored at −80 °C until DNA extraction. Within 30 min after slaughter, we collected a *longissimus dorsi* muscle sample (approximately 200 g per pig, stored at −20 °C) from each pig to determine the amino acid and fatty acid contents. Some samples were excluded from analyses of amino acids and fatty acids owing to quality issues.

### 2.2 Determination of amino acid content

To evaluate amino acids, we adopted an analytical approach based on liquid chromatography-tandem mass spectrometry (LC-MS/MS). Samples were first thawed. An aliquot of 0.05 g was mixed with 500 μl of 70% methanol (Merck, Darmstadt, Germany)/water (Millipore, Bradford, USA), vortexed at 2,500 *r*/min for 3 min, and centrifuged at 12,000 *r*/min for 10 min at 4 °C. The supernatant (300 μl) was stored at −20 °C for 30 min and then recentrifuged under the same conditions. Finally, 200 μl of the supernatant was passed through a Protein Precipitation Plate for LC-MS/MS (21). Analyses were performed on an LC-ESI-MS/MS system (UPLC: ExionLC AD; MS: QTRAP^®^ 6500+ System). Chromatographic conditions were as follows: column, ACQUITY BEH Amide (2.1 × 100 mm, 1.7 μm); mobile phase A [water with 2 mM ammonium acetate (Sigma-Aldrich, St. Louis, MO, USA) and 0.04% formic acid (Sigma-Aldrich, St. Louis, MO, USA)] and B [acetonitrile (Merck, Darmstadt, Germany) with 2 mM ammonium acetate and 0.04% formic acid]; gradient program 90% B (0–1.2 min), 60% B (9 min), 40% B (10–11 min), 90% B (11.01–15 min); flow rate, 0.4 ml/min; column temperature, 40 °C; injection volume, 2 μl. Mass spectrometry was operated in both positive and negative ion modes with an ESI Turbo Ion-Spray interface: source temperature, 550 °C; ion spray voltage, 5,500 V (positive) and −4,500 V (negative); curtain gas, 35.0 psi. MRM transitions were monitored according to the elution time of target amino acids. Qualitative analyses were conducted using the Metware Database based on authentic standards. External standard curves were prepared by diluting stock solutions (1 mg/ml in methanol) to a series of concentrations (10–20,000 ng/ml). The amino acid content (ng/g) was calculated as follows:


Amino acid content (ng/g)=c×V/1,000/m

where c is the concentration (ng/ml) obtained by substituting the integrated peak area ratio of the sample into the standard curve, V is the volume of the solution used for extraction (μl), and m is the mass of the weighed sample (g).

### 2.3 Determination of fatty acid content

We adopted an analytical method based on the GC-MS/MS platform to quantitatively analyze fatty acid contents. After thawing, 0.05 g of sample was mixed with 150 μl of methanol (Merck, Darmstadt, Germany), 200 μl of methyl tert-butyl ether (Merck, Darmstadt, Germany), and 50 μl of 36% phosphoric acid (Sigma-Aldrich, St. Louis, MO, USA), pre-cooled to −2 °C. The mixture was vortexed (2,500 rpm, 3 min) and centrifuged (12,000 rpm, 5 min, 4 °C). Then, 200 μl of the supernatant was transferred to a new tube. After drying the 200 μl supernatant under N_2_, the residue was reconstituted in 300 μl of 15% boron trifluoride-methanol (RHAWN, Shanghai, China), vortexed for 3 min at 2,500 rpm, and derivatized at 60 °C for 30 min. Samples were cooled to room temperature, and 500 μl of n-hexane (Merck, Darmstadt, Germany) and 200 μl of saturated NaCl solution (Sigma-Aldrich, St. Louis, MO, USA) were added. Following vortexing (3 min) and centrifugation (12,000 rpm, 5 min, 4 °C), 100 μl of the n-hexane layer solution was collected for the GC-MS analysis (22, 23). Derivatized samples were analyzed using an Agilent 8890 GC/5977B MS system equipped with a DB-5MS capillary column (30 m × 0.25 mm × 0.25 μm; Agilent). High-purity helium (>99.999%) was used as carrier gas at 1.0 ml/min. The oven temperature program was as follows: 40 °C (hold for 2 min), 200 °C at 30 °C/min (hold for 1 min), 240 °C at 10 °C/min (hold for 1 min), 285 °C at 5 °C/min (hold for 3 min). Injection parameters were as follows: 230 °C inlet temperature, 1.0 μl splitless mode. MS conditions were as follows: EI source (70 eV); ion source temperature 230 °C; transfer line 240 °C; quadrupole 150 °C; solvent delay 4 min; selected ion monitoring (SIM) mode. Standard stock solutions (1 mg/ml in MTBE, Merck, Darmstadt, Germany) were stored at −20 °C and serially diluted to prepare calibration standards (0.01–50 μg/ml). Peak areas were quantified using Agilent MassHunter software. The absolute fatty acid content (μg/g) was calculated as follows:


$$Fatty\;acid\;content\left(ug/g\right):=c\times V_{3}/1,000\times V_{1}/V_{2}/m$$

where c is the concentration (μg/ml) derived by substituting the integrated peak area of the sample into the standard curve, V_1_ is the volume of the sample extraction solution (μl), V_2_ is the volume of the collected supernatant (μl), V_3_ is the reconstitution volume (μl), and m is the mass of the weighed sample (g).

### 2.4 Statistical analyses of phenotypes

Mean values and standard errors of the mean (SEM) for each trait were calculated and compared among populations using one-way analysis of variance (ANOVA) followed by Bonferroni *post-hoc* tests in SPSS 27.0 for Windows. Relationships between meat quality traits were assessed using Spearman's rank correlation coefficients in SPSS.

### 2.5 Whole-genome sequencing

Genomic DNA was extracted from ear tissue, and the purity and integrity of the DNA were assessed. Specifically, DNA purity was evaluated with a Nanodrop spectrophotometer by quantifying A260/A280 ratios (1.8–2.0). DNA integrity was verified via agarose gel electrophoresis. Then, the DNA sample was fragmented by sonication. The DNA fragments underwent end repair, A-tailing, and ligation of index adapters, followed by PCR amplification. The constructed libraries were evaluated with respect to quality and yield. After the libraries passed quality control, they were pooled based on their effective concentrations and the target output requirement. Finally, DNA sequencing libraries for all samples were sequenced using the Illumina platform (Novogene Bioinformatics Technology Co., Ltd., Beijing, China) to obtain 150 bp (PE150) paired-end reads.

### 2.6 Data processing

To obtain clean data, low-quality and adapter-contaminated reads were removed. To detect genetic variants in the pig genomes, the clean reads were aligned to the *Sscrofa*11.1 (GCF_000003025.6) reference genome using bwa-mem2 software (v2.2.1), and aligned reads were sorted by reference genome coordinates using SAMtools (v1.17). Subsequently, duplicated reads were removed using sambamba (v0.6.6) with the “markdup” parameter to avoid overestimation of the sequencing depth and false-positive variants introduced during PCR amplification. SNP calling was performed using GATK (v4.5.0.0) to generate a GVCF file, followed by filtering to retain high-quality SNPs using the same software. The GATK VariantFiltration module was employed with the following parameters: QD <2.0, MQ <40.0, FS > 60.0, MQRankSum <−12.5, and ReadPosRankSum <−8.0. For SNP quality control (QC), additional filtering was performed using VCFtools (v0.1.17) as follows: removal of loci with quality scores <Q20, missing rate > 0.1, minor allele frequency (MAF) <0.05, or individual read depth (DP) <3 as well as multi-allelic loci (retaining only biallelic SNPs). SNPs retained after filtering were used for subsequent analyses. SNP annotation was conducted using ANNOVAR (2013-06-21) (24).

### 2.7 PCA

A principal component analysis (PCA) was performed using GCTA (v1.94.1) to evaluate high-quality SNPs obtained from 73 pigs across three distinct populations (25). Analyses were restricted to autosomal loci, with multiallelic variants and genotyping mismatches excluded prior to computation. PCA results were visualized using the ggplot2 package in R (v4.3.1).

### 2.8 Linkage disequilibrium analysis

Linkage disequilibrium (LD) values for each population were calculated based on the squared correlation coefficient (*r*^2^) between each pair of SNPs within 100 kb windows using PopLDdecay (v3.41) software with default parameters (26). The relationship between LD decay and physical distance among SNPs was visualized using PopLDdecay.

### 2.9 Genome-wide association analysis

To test the effects of each variant on meat quality traits, we performed a GWAS using GEMMA (v0.98.3) with the joint dataset covering all phenotypes and matched genotypes (27). For GWAS analysis, we employed the linear mixed model in GEMMA to correct for population stratification and individual relatedness. The model is as follows:


y=Xα+Zβ+Wμ+e

In the formula, y is the vector of phenotype values; Z is genotype matrix; X is the covariate matrix, including the fixed effect of sex and the first three principal components of the PCA; α is a vector of the covariate coefficients, including the intercept; W is the genetic relationship matrix between individuals calculated using GCTA; β is the vector of SNP fixed effects; μ is the random genetic effects, accounting for individual relatedness; and e is the vector of residual errors.

This GWAS aimed to identify genetic loci associated with amino acid and fatty acid contents in the *longissimus dorsi* muscle of each pig. The R package (v3.5.1) was used to generate Manhattan plots and QQ plots. A significance threshold of –Log_10_ (*P*) ≥ 6 (i.e., *P* ≤ 1 × 10^−6^) was used to identify significant SNPs. QQ plots were used to assess whether false-positive SNPs were introduced by population stratification.

### 2.10 SNP annotation and functional analysis

SNPs above the genome-wide significance threshold (*P* = 1 × 10^−6^) were defined as significant. Genes located within 40 kb upstream or downstream of these significant SNPs were annotated as candidate genes for the target traits. To explore the potential biological functions of the candidate genes, functional annotation focusing on Gene Ontology (GO) biological processes (BP) was conducted using Metascape (28, 29). Furthermore, the phenotypes of individuals with different genotypes at the significant SNPs were analyzed. Independent *t*-tests were used for comparisons between two genotypes, and one-way analysis of variance (ANOVA) and then Bonferroni *post-hoc* tests were applied for comparisons among three genotypes using SPSS 27.0. Graphs were drawn using GraphPad Prism (v8.0.2). To validate the potential functions of candidate genes, we used pig RNA-Seq data from the PIGOME database and performed a differential expression analysis of candidate genes across different tissues (30).

## 3 Results

### 3.1 Phenotypic analysis of three crossbred pig populations and phenotypic correlations

Crossbred pigs are widely used in pig production systems. To assess meat quality, we collected *longissimus dorsi* muscle from 49 pigs from three two-way crossbred populations (YT, *n* = 10; YN, *n* = 20; DT, *n* = 19). In total, 17 amino acids [including eight essential amino acids (EAAs) for humans, four flavor amino acids (FAAs), and five additional amino acids] as well as 42 fatty acids [including 18 saturated fatty acids (SFAs), 11 monounsaturated fatty acids, and 13 polyunsaturated fatty acids] were determined.

For the composition of amino acids in the *longissimus dorsi* muscle of 49 pigs, we compared the total amino acid (TAA) content across three pig populations (Table 1). The TAA content of DT pigs was significantly lower than those of the YT and YN pigs (*P* < 0.05). There were no significant differences in the total EAA content among the three populations; however, there were differences in single EAAs. For three of the seven EAAs with differences in content among populations, the DT population displayed the lowest levels (*P* < 0.05). Notably, the tryptophan (Trp) content in the DT population was 3.44 times greater than that in the YT population and 1.53 times greater than that in the YN population (*P* < 0.05). This elevated Trp content may partially compensate for the low total EAA content in the DT population. Additionally, YT pigs had a significantly higher total FAA content compared with YN and DT populations (*P* < 0.05). In particular, the alanine (Ala) content in the YT population was 2.40 times higher than that in YN pigs and 2.30 times higher than that in DT pigs (*P* < 0.05). Only one FAA (glycine, Gly) differed significantly between the YN and DT populations (*P* < 0.05), with higher levels in YN pigs than in DT pigs (*P* < 0.05). These findings indicate obvious differences in meat flavor among the three crossbred pig populations. The lower FAA content in DT than in YT and YN implies that Yorkshire as the sire breed may contribute to higher FAA contents than those for Duroc as the sire breed, thereby enhancing pork flavor.

**Table 1:** Contents of selected amino acids (ng/g) in the *longissimus dorsi* muscle tissue from three crossbred pig populations (*n* = 49).

**Amino acid**	**YT (*n* = 10)**	**YN (*n* = 20)**	**DT (*n* = 19)**
Isoleucine (Ile)	42,040.15 ± 1,734.01^a^	41,339.48 ± 1,475.02^a^	32,923.28 ± 2,443.86^b^
Leucine (Leu)	57,062.85 ± 2,498.22^b^	70,643.01 ± 2,020.62^a^	46,584.97 ± 4,536.97^b^
Lysine (Lys)	31,809.01 ± 2,349.56^a^	20,504.84 ± 771.46^b^	17,670.02 ± 1,121.19^b^
Phenylalanine (Phe)	18,646.14 ± 721.03^ab^	20,523.26 ± 569.35^a^	17,880.98 ± 895.04^b^
Threonine (Thr)	41,481.67 ± 1,990.36	39,081.88 ± 1,092.85	39,542.18 ± 2,963.58
Valine (Val)	50,577.47 ± 2,791.73^ab^	52,860.91 ± 1,528.73^a^	41,791.11 ± 3,031.69^b^
Methionine (Met)	7,231.65 ± 432.37^ab^	5,427.27 ± 254.45^b^	8,534.07 ± 1,183.98^a^
Tryptophan (Trp)	14,641.56 ± 557.65^c^	32,813.82 ± 912.27^b^	50,356.87 ± 4,518.21^a^
∑Essential amino acid (EAA)	263,490.5 ± 11358.72	283,194.47 ± 7,035.16	255,283.48 ± 13,172.83
Glutamic acid (Glu)	184,341.23 ± 22,844.33^b^	323,710.32 ± 23,916.79^a^	290,762.26 ± 36,374.33^ab^
Aspartic acid (Asp)	10,664.89 ± 1,126.5^b^	24,560.27 ± 2,998.92^ab^	29,086.02 ± 4,697.58^a^
Glycine (Gly)	111,519.46 ± 4,437.61^b^	165,315.19 ± 5,208.12^a^	113,400.64 ± 10,770.5^b^
Alanine (Ala)	1,240,997.64 ± 18,238.08^a^	516,622.98 ± 8,706.58^b^	538,752.89 ± 8,480.1^b^
∑Flavor amino acid (FAA)	1,547,523.21 ± 31,811.47^a^	1,030,208.76 ± 24,919.75^b^	972,001.81 ± 42,771.93^b^
∑Total amino acid (TAA)	2,021,451.52 ± 44,000.61^a^	1,862,324.85 ± 28,036.76^a^	1,548,979.45 ± 89,279.76^b^

Values in the same row with different letters differ significantly at *P* < 0.05. The data are presented as the mean ± standard error of the mean (SEM). Essential amino acids (EAA) = Ile + Leu + Lys + Phe + Thr + Val + Met + Trp. Flavor amino acids (FAA) = Glu + Asp + Gly + Ala. Total amino acids (TAA) = Ile + Leu + Lys + Phe + Thr + Val + Met + Trp + Glu + Asp + Gly + Ala + Tyr + Ser + His + Pro + Arg.

For fatty acid contents in the *longissimus dorsi* muscle, one sample was lost during the assay, so we analyzed the fatty acid composition across three populations based on data from a total of 48 pigs (Table 2). Contrary to the above observation that DT pigs exhibit low amino acid contents, these pigs generally exhibited the highest contents of most detected fatty acids among populations. The total fatty acid (TFA) content was highest in the *longissimus dorsi* muscle of DT pigs, followed by YN pigs and YT pigs (*P* < 0.05). Similar results were observed for the unsaturated fatty acid (UFAs) and SFAs. The contents of single fatty acids were generally highest in DT pigs. For example, the content of palmitic acid (C16:0) in DT pigs was 1.47 times higher than that in YT pigs and 1.33 times higher than that in YN pigs; this fatty acid is closely linked to the nutritional value and processing properties of pork. The oleic acid (C18:1n9c) content in DT pigs was more than 1.50 times those in YT and YN pigs; this fatty acid contributes to the flavor and nutritional value of pork.

**Table 2:** Contents of selected fatty acids (μg/g) in the *longissimus dorsi* muscle tissue from three crossbred pig populations (*n* = 48).

**Fatty acid**	**YT (*n* = 10)**	**YN (*n* = 20)**	**DT (*n* = 18)**
Myristoleic acid (C14:0)	1.17 ± 0.01^b^	1.54 ± 0.04^a^	1.48 ± 0.05^a^
Palmitic acid (C16:0)	364.10 ± 7.74^b^	400.98 ± 6.23^b^	534.19 ± 30.82^a^
Stearic acid (C18:0)	257.20 ± 7.05^b^	287.76 ± 4.48^b^	346.48 ± 14.01^a^
cis-9-palmitoleic acid (C16:1)	27.25 ± 0.80^b^	30.45 ± 1.14^b^	42.79 ± 3.59^a^
Oleic acid (C18:1n9c)	258.31 ± 7.78^b^	219.09 ± 3.76^b^	407.83 ± 42.53^a^
linoleic acid (C18:2n6c)	356.44 ± 11.84^b^	468.38 ± 9.17^a^	488.46 ± 13.46^a^
α-linolenic acid (C18:3n3)	5.71 ± 0.15^c^	16.10 ± 0.33^a^	12.81 ± 0.74^b^
γ-linolenic acid (C18:3n6)	5.25 ± 0.07	5.23 ± 0.17	5.06 ± 0.18
Eicosapentaenoic acid (C20:5n3)	11.23 ± 0.17^b^	22.93 ± 0.68^a^	20.40 ± 0.94^a^
∑Saturated fatty acid (SFA)	684.99 ± 13.28^b^	729.47 ± 10.82^b^	928.87 ± 46.17^a^
∑Monounsaturated fatty acid (MUFA)	361.78 ± 8.88^b^	356.44 ± 5.38^b^	605.33 ± 56.79^a^
∑Polyunsaturated fatty acid (PUFA)	583.39 ± 16.36^b^	888.43 ± 17.76^a^	864.55 ± 24.13^a^
∑Unsaturated fatty acid (UFA)	945.17 ± 23.46^c^	1,244.87 ± 22.03^b^	1,469.88 ± 60.38^a^
∑Total fatty acid (TFA)	1,630.15 ± 35.52^c^	1,974.35 ± 32.52^b^	2,398.75 ± 104.14^a^

Values in the same row with different letters differ significantly at *P* < 0.05. The data are presented as the mean ± SEM. EPA, Eicosapentaenoic acid; SFA, saturated fatty acid; UFA, unsaturated fatty acid. Total fatty acid (TFA) = SFA + UFA.

**Figure 1:**
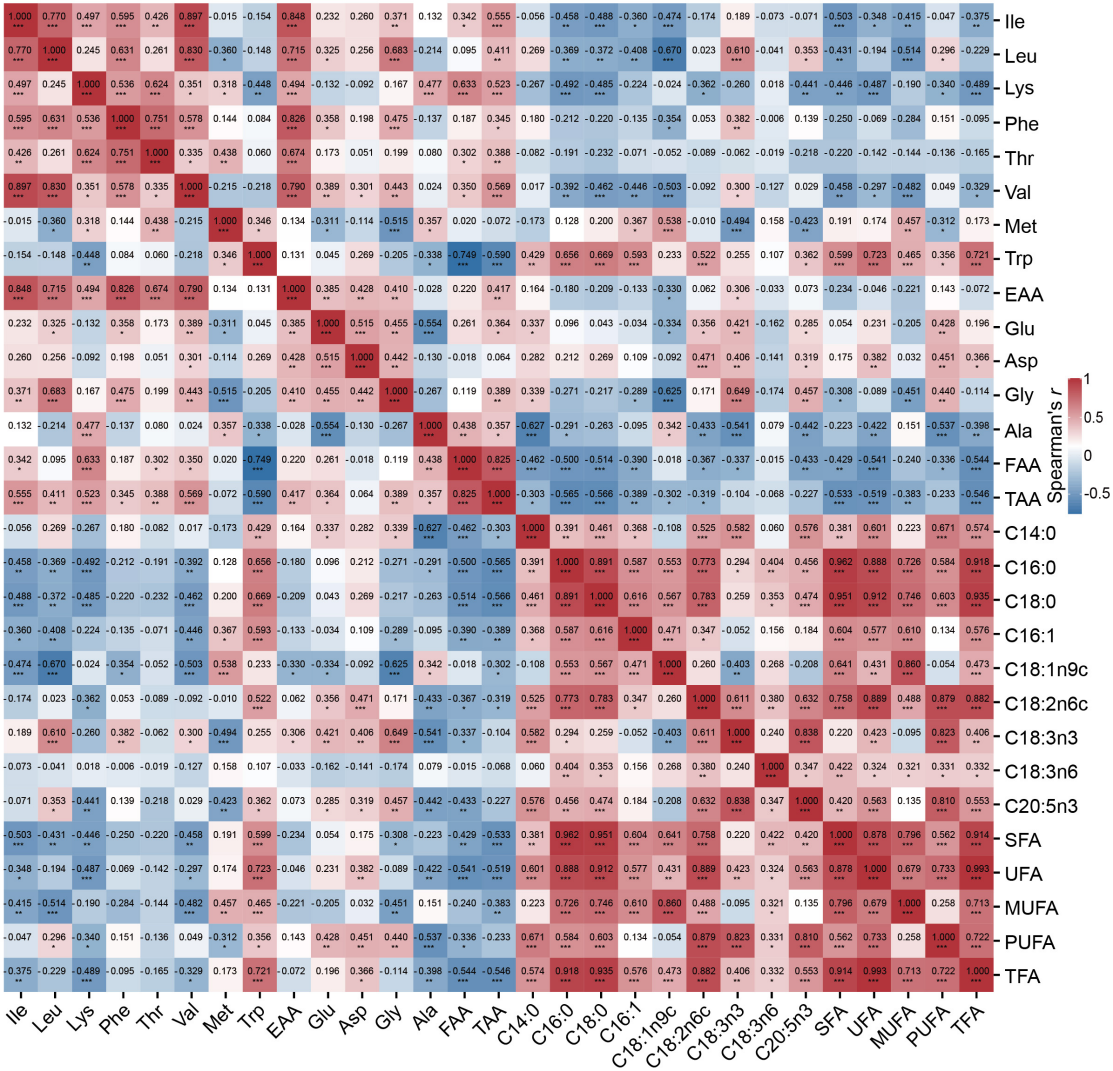
Heatmap of the Spearman correlation coefficients for relationships between fatty acid and amino acid contents in the *longissimus dorsi* muscle tissue of three different two-way crossbred pigs (*n* = 48). *, **, and *** represent significance levels of 0.05, 0.01, and 0.001, respectively.

We next integrated data for the three populations to explore associations between metabolite abundance (i.e., amino acids and fatty acids) in the *longissimus dorsi* muscle of pigs (Figure 1). The majority of amino acids were positively correlated with one another, including EAAs and FAAs. Notably, methionine (Met) and tryptophan (Trp) were negatively correlated with other amino acids. For instance, the correlation coefficients for Met with leucine (Leu), glycine (Gly), and glutamic acid (Glu) were −0.360 (*P* < 0.05), −0.311 (*P* < 0.05), and −0.515 (*P* < 0.001), respectively. Fatty acids also generally displayed positive correlations with each other. However, oleic acid (C18:1n9c) was significantly negatively correlated with α-linolenic acid (C18:3n3, −0.403, *P* < 0.01). Furthermore, the correlations between most amino acids and fatty acids were generally weak (mean = −0.09) and predominantly negative (64.76%), with 75% of correlation coefficients ranging from −0.67 to 0.16 (i.e., the minimum value was −0.67 and Q3, the third quartile, was 0.16). Of note, the Trp content was positively associated with the contents of three fatty acids: palmitic acid (C16:0, 0.656, *P* < 0.001), stearic acid (C18:0, 0.669, *P* < 0.001), and linoleic acid (C18:2n6c, 0.552, *P* < 0.001). Additionally, there was a positive correlation between glycine and α-linolenic acid contents (0.649, *P* < 0.001). These findings suggest potential avenues for improving meat quality in pig breeding.

### 3.2 Detection of SNPs in three crossbred pig populations

**Figure 2:**
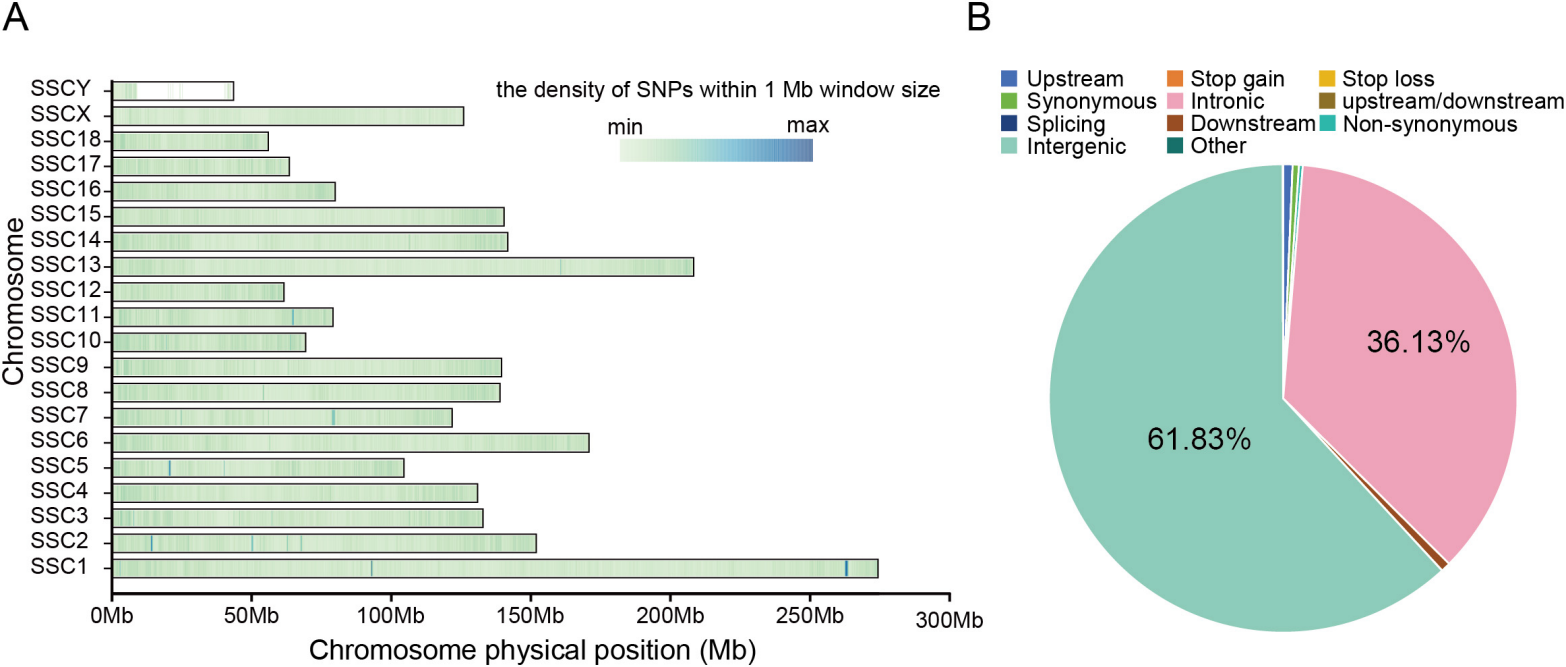
Distribution of SNPs across the chromosomes detected in pigs from admixed populations. **(A)** Genome-wide distribution of detected SNPs on chromosomes. Calculated as the number of SNPs per 1 Mb. **(B)** Distribution of detected SNPs in different genomic elements. Upstream regions were defined as the 1 kb region upstream from the gene start site. Downstream regions were defined as the 1 kb region of the gene end site. Upstream/Downstream indicates a variant located in the 1 kb upstream region of one gene and in the 1 kb downstream region of another gene.

To further explore the genetic basis of the amino acid and fatty acid compositions in the *longissimus dorsi* muscle, we performed whole-genome resequencing of 73 pigs (YN, *n* = 20; DT, *n* = 20; YT, *n* = 33) from three crossbred populations (Supplementary Table S1). In total, 3.5362 Terabase (Tb) of raw data were generated from 73 samples. After filtering, 3.5025 Tb of clean data were retained with an average depth of approximately 20 × . Combined with the pig reference genome (*Sscrofa*11.1) from NCBI, we obtained 24,125,658 SNPs after applying QC filters. These SNPs were evenly distributed across the chromosomes of the pig genome (Figure 2, Supplementary Table S2), and most SNPs were located in intronic (36.13%) and intergenic regions (61.83%). Additionally, base transitions accounted for the majority of SNPs at 70.89% (17,101,678), while transversions accounted for 29.11% (7,023,980).

### 3.3 Principal component analysis and linkage disequilibrium

**Figure 3:**
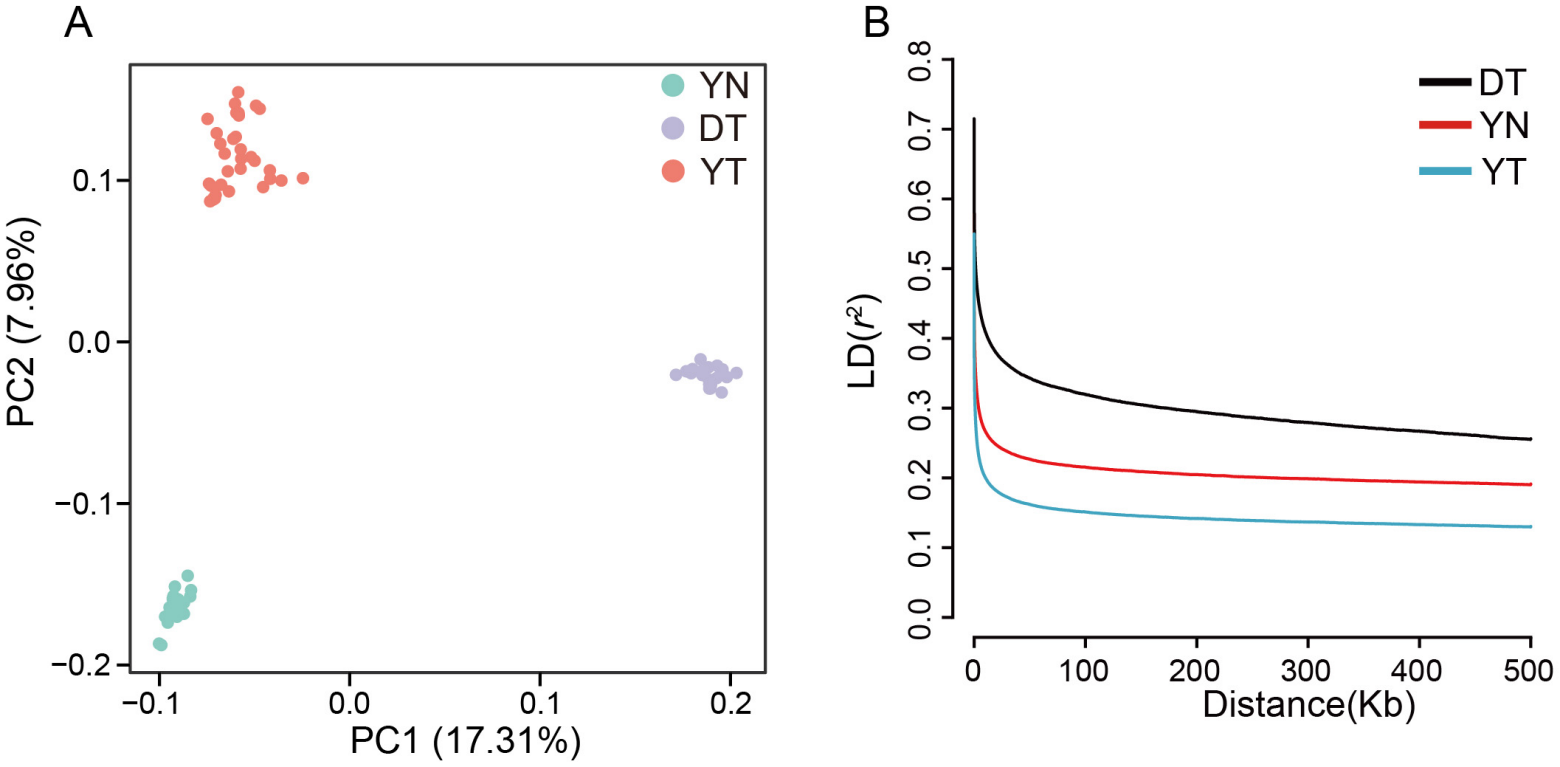
Population structure of 73 admixed pigs and extent of LD (*r*^2^) as a function of physical distance between SNPs. **(A)** PCA plots for the YN (green), DT (purple), and YT (red) samples based on the genotyped SNPs. **(B)** LD analysis using samples from YN (*n* = 20), DT (*n* = 20), and YT (*n* = 33). The *x*-axis represents the physical distance between SNPs (kilobases, kb) and the *y*-axis indicates the strength of LD (measured by *r*^2^).

To evaluate genetic differentiation, we performed a PCA based on 24,125,658 high-quality SNPs (Figure 3A). The pigs were clearly clustered into three populations by the first (PC1) and second principal components (PC2), with PC1 and PC2 accounting for 17.31% and 7.96% of the total variance, respectively. These clusters were consistent with the three populations documented at sample collection, confirming the accuracy of population classification.

To estimate the extent of LD in the three pig populations, we computed the squared correlation coefficient (*r*^2^) between SNP pairs (Figure 3B). LD levels declined rapidly with increasing physical distance between SNPs (Figure 3B), with obvious differences in LD decay rates among the three populations. At the same physical distance (100 kb), the LD decay rates of the three populations tended to stabilize, and *r*^2^ values were highest in the DT population, followed by the YN population and the YT population. These results indicate that DT pigs have lower genetic diversity (reflected by higher *r*^2^) than those of other two pig populations.

### 3.4 Genome-wide association analysis of the three crossbred pig populations

**Figure 4:**
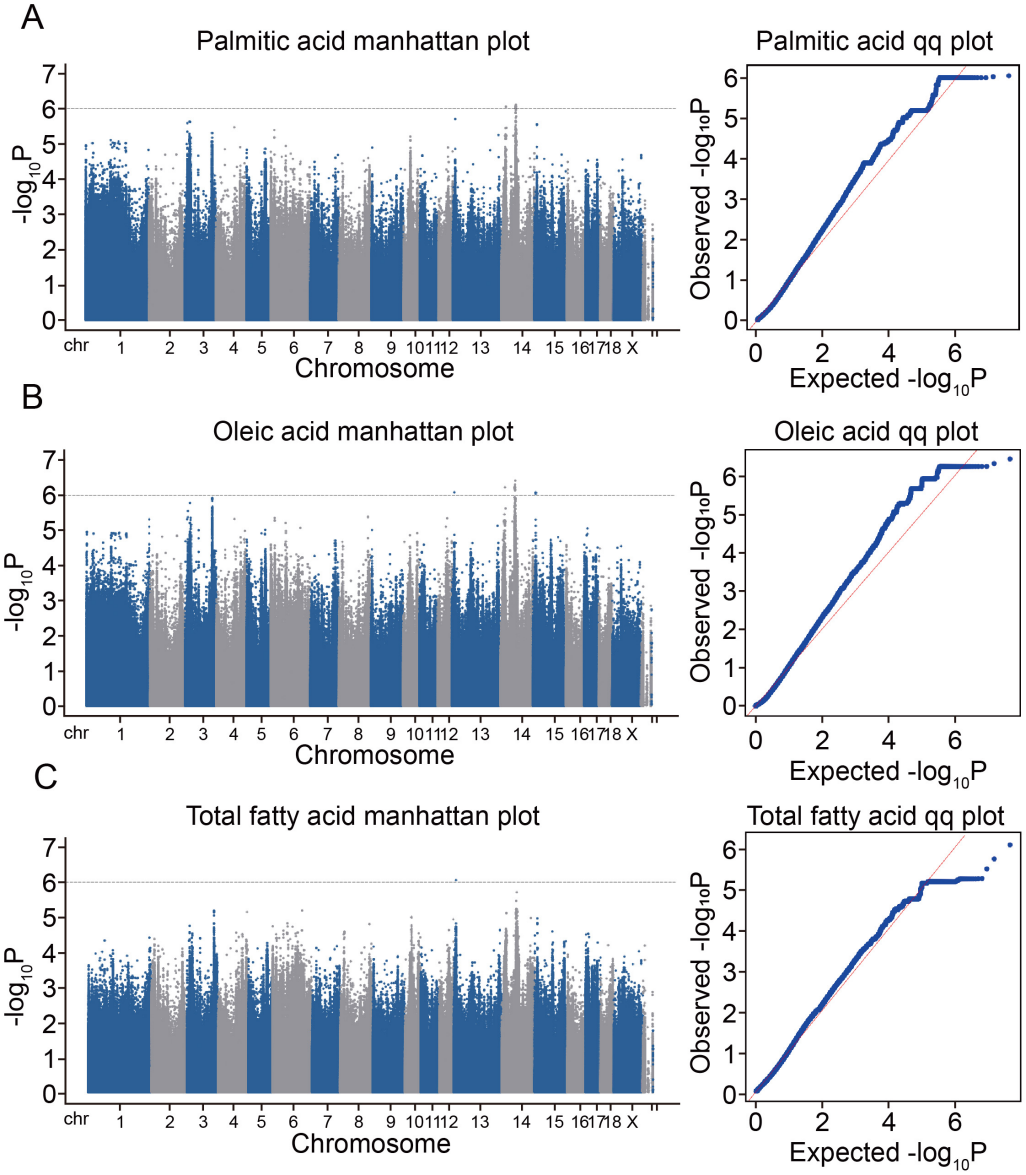
Manhattan and QQ Plots of Significant SNPs from the GWAS in an Admixed Population (*n* = 73). **(A)** Manhattan plot and QQ plot of total palmitic acid. **(B)** Manhattan plot and QQ plot of oleic acid. **(C)** Manhattan plot and QQ plot of total fatty acid. In the Manhattan plots, the dotted horizontal lines show the suggestive significance levels. The dots above the dotted line of the Manhattan plot are significant SNPs. The quantile-quantile plots show the late separation between observed and expected values.

To identify SNPs associated with amino acid and fatty acid contents in pork, we conducted a GWAS using phenotypic and resequencing data for 49 crossbred pigs (Figure 4). The GWAS model was validated via QQ plots, which showed early consistency between observed and expected *P*-values and late mild separation (Figure 4). In the GWAS, 146 SNPs (Supplementary Table S3) were identified at the significance threshold (*P* = 1 × 10^−6^); no significant SNPs associated with amino acid contents were detected in this study. Based on the *Sus scrofa* 11.1 reference genome, we annotated 19 candidate genes (*ZNF37A, ABCB10, GALNT2, RHOBTB1, BICC1, RET, HNRNPF, TAF5L, TMEM26, URB2, FXYD4, PHYHIPL, FAM13C, LRP1B, RHOU, PGBD5, LOC110256649, LOC110256821*, and *UBE2E2*) within 40 kb of the 146 significant SNPs. Among the SNPs, 68, 77 and 1 were associated with palmitic acid, oleic acid, and TFA, respectively. Specifically, 68 palmitic acid-related SNPs were localized on *Sus scrofa* chromosome 14 (SSC14), involving 15 genes; 77 oleic acid-related SNPs were distributed across SSC13, SSC14, and SSC15, associated with 19 genes; and one TFA-related SNP was localized on SSC13, involving one gene. Notably, the SNPs associated with different fatty acid traits (i.e., palmitic acid, oleic acid, and TFA) exhibited a close association. Specifically, 68 SNPs were shared between palmitic acid and oleic acid contents, eight SNPs were specifically related to the oleic acid content, and one SNP overlapped between oleic acid and TFA.

### 3.5 Functional annotation of candidate genes related to fatty acid content

We performed functional annotation of 19 candidate genes focusing on the BP category using Metascape (Supplementary Table S4). Functional annotation revealed 14 candidate genes with clear biological functions, and five (*TMEM26, PHYHIPL, FAM13C, LOC110256649*, and *LOC110256821*) were excluded owing to undefined functions (i.e., functional annotation results were categorized as “none” in Metascape; Supplementary Table S4). Four candidate genes had functional annotations indicating potential associations with the fatty acid content. *ABCB10* is involved in export from the mitochondrion (GO:0170037), mitochondrial unfolded protein response (GO:0034514), and positive regulation of heme biosynthetic process (GO:0070455). *GALNT2* mediates protein *O*-linked glycosylation via serine (GO:018242), protein *O*-linked glycosylation via threonine (GO:0018243), and peptidyl-threonine modification (GO:0018210). *RET* regulates posterior midgut development (GO:0007497), Peyer's patch morphogenesis (GO:0061146), and positive regulation of metanephros development (GO:0072216). *LRP1B* is associated with receptor-mediated endocytosis (GO:0006898), endocytosis (GO:0006897), and import into cell (GO:0098657). These BP terms indicate that the candidate genes affect fatty acid metabolism in pork through distinct pathways.

**Figure 5:**
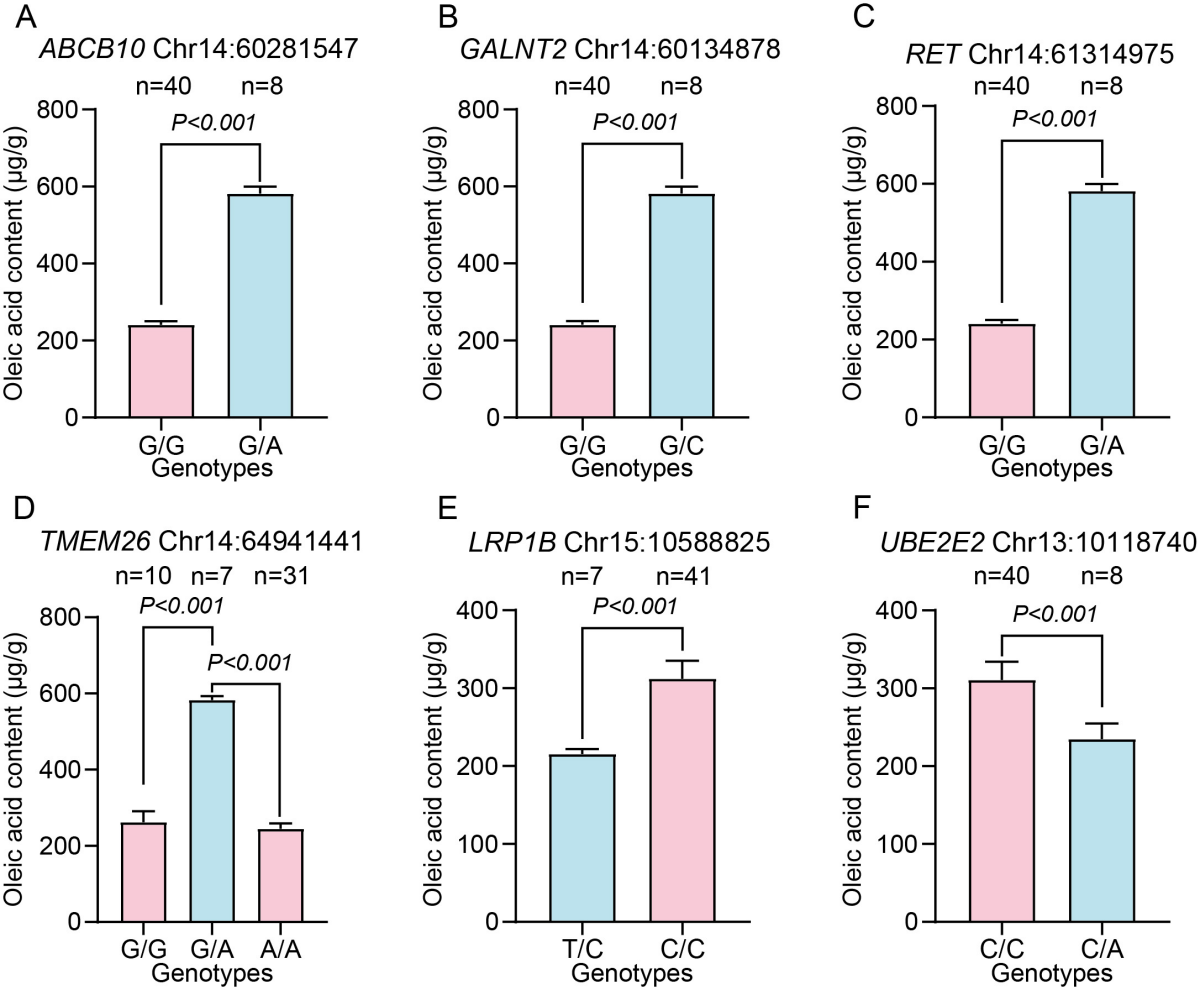
Effects of six candidate genes on oleic acid contents in *longissimus dorsi* muscle of pigs mediated by different genotypes. The *y*-axis represents the oleic acid contents in *longissimus dorsi* muscle of pigs, while different groups on the *x*-axis indicate distinct genotypes, with pink representing homozygous and blue representing heterozygous alleles. Bars represent means ± SEM. **(A–F)** Differences in the oleic acid contents of *longissimus dorsi* muscle among individuals with different genotypes of SNPs associated with *ABCB10, GALNT2, RET, TMEM26, LRP1B*, and *UBE2E2*, respectively. **(A)** Oleic acid contents for the SNP at Chr14:60281547 (G > A). **(B)** Oleic acid content for the SNP at Chr14:60134878 (G > C). **(C)** Oleic acid content for the SNP at Chr14:61314975 (G > A). **(D)** Oleic acid content for the SNP at Chr14:64941441 (G > A). **(E)** Oleic acid content for the SNP at Chr15:10588825 (T > C). **(F)** Oleic acid content for the SNP at Chr13:10118740 (C > A).

Further literature mining identified six candidate genes related to fatty acid metabolism, namely *ABCB10* (31, 32), *GALNT2* (33–36), *RET* (37), *TMEM26* (38–42), *LRP1B* (43–45), and *UBE2E2* (46–48). For these six candidate genes, we analyzed the phenotypes of individuals with different genotypes at the six most significant SNPs, which showed the most significant associations (i.e., smallest *P*-values) in GWAS and resided within 40 kb flanking regions of their corresponding candidate genes (Figure 5). For the same gene, individuals with different genotypes exhibited significant differences in oleic acid content (Figures 5A–F, *P* < 0.01). Notably, for the most significant SNPs for *ABCB10, RET, GALNT2*, and *TMEM26*, the oleic acid content was higher in heterozygous than in homozygous individuals (Figures 5A–D, *P* < 0.001). In contrast, for significant SNPs for *LRP1B* and *UBE2E2*, the oleic acid content was higher in homozygous than in heterozygous individuals (Figures 5E, F, *P* < 0.001). Specifically, at loci Chr14:60281547 (*ABCB10*) and Chr14:61314975 (*RET*), pigs with the G/A genotype had significantly higher oleic acid contents than those of pigs with the G/G genotype (Figures 5A, C, *P* < 0.001). At Chr13:10118740 (*UBE2E2*), the C/C genotype group had a significantly higher fatty acid content than that of the C/A genotype group (Figure 5F, *P* < 0.001).

**Figure 6:**
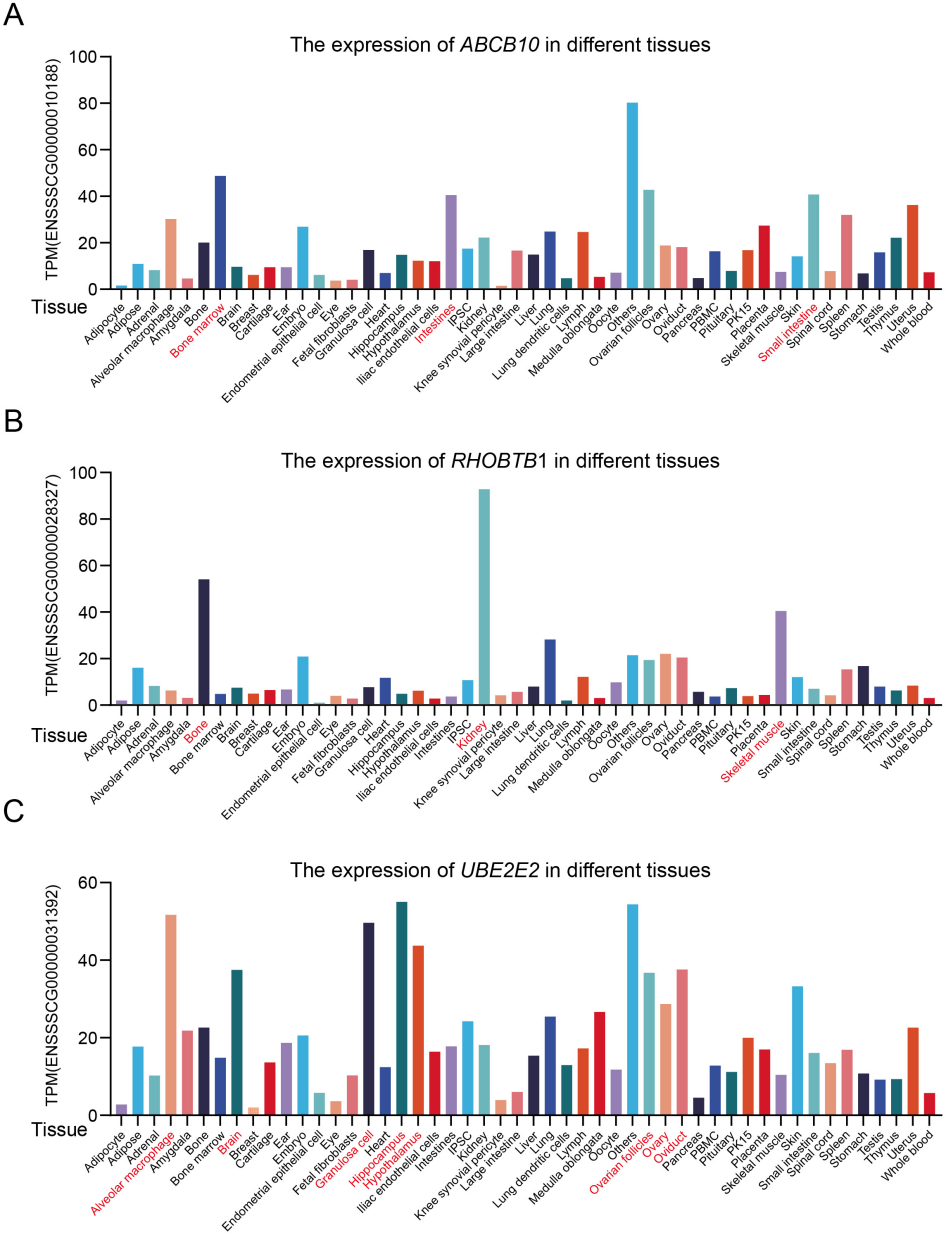
Expression levels of *ABCB10, RHOBTB1* and *UBE2E2* in different tissues were retrieved from the public PIGOME database (30). TPM, transcripts per kilobase of exon model per million mapped reads. **(A)** Expression of *ABCB10* in various tissues. **(B)** Expression of *RHOBTB1* in various tissues. **(C)** Expression of *UBE2E2* in various tissues.

To further explore the potential functions of candidate genes, we integrated pig RNA-Seq data from the PIGOME database and analyzed the transcriptional characteristics of *ABCB10, RHOBTB1* and *UBE2E2* in different tissues (Figure 6). *ABCB10* was highly expressed in the bone marrow, intestines, and small intestine (Figure 6A). *RHOBTB1* showed notable expression changes in bone, kidney, and skeletal muscle (Figure 6B). *UBE2E2* was abundantly expressed in the alveolar macrophages, brain, granulosa cells, hippocampus, hypothalamus, ovarian follicles, ovary, and oviduct (Figure 6C). The differential expression levels of candidate genes (*ABCB10, RHOBTB1*, and *UBE2E2*) further indicate that they may be involved in the fat metabolism process through different pathways.

## 4 Discussion

Crossbreeding Western pig breeds with Chinese indigenous pigs is a widely used and effective strategy for improving meat quality while retaining production performance (49, 50). Amino acids are key determinants of meat flavor (e.g., Glu and Asp confer an umami taste) and play a pivotal role in assessing the nutritional quality of pork. The YN population had a significantly higher Glu content than that of the YT population (*P* < 0.05), contributing to a more intense umami flavor in its pork. Alanine (Ala), contributing to sweetness, was most abundant in the YT population (*P* < 0.05). Additionally, the nutritional value of pork protein primarily depends on the type and quantity of EAAs. As a key precursor for the synthesis of serotonin and melatonin in humans, the Trp content of the DT population was higher than those of YT and YN populations (*P* < 0.05), facilitating human absorption and utilization of this EAA. Notably, the DT population generally had higher fatty acid contents (e.g., SFA and UFA) than YT and YN populations (*P* < 0.05), and appropriate levels of SFAs and UFAs are beneficial for pork quality (e.g., tenderness, juiciness, and flavor) and nutritional value. Correlation analyses also revealed positive associations between key flavor amino acids (e.g., glutamate, aspartate) and polyunsaturated fatty acids (*r* > 0.4, *P* < 0.01). These associations provide a basis for breeding pigs with tailored nutritional and taste qualities (51).

In our GWAS, we identified 146 significant SNPs and 19 candidate genes. Notably, some SNPs were consistently associated with different fatty acid traits (palmitic acid, oleic acid, and TFA), suggesting that they participate in the shared genetic regulation of these traits. Six genes (*ABCB10, GALNT2, RET, TMEM26, LRP1B*, and *UBE2E2*) were closely associated with fatty acid metabolism and were involved in related biological processes, such as lipid metabolism, glucose homeostasis, and energy balance. *ABCB10* regulates mitochondrial bilirubin levels to influence mitochondrial function, which in turn affects fatty acid oxidation (31, 32). *GALNT2* encodes a glycosyltransferase that modulates lipid metabolism via *O*-glycosylation. Loss-of-function variants in *GALNT2* reduce high-density lipoprotein cholesterol (HDL-C) levels, while the overexpression of this gene impairs pancreatic function (33–36). *RET* regulates the secretion of glucagon-like peptide-1 (GLP-1) and peptide YY (PYY) (37). These two hormones modulate gut motility and nutrient absorption, thereby impacting glucose homeostasis. *TMEM26* encodes a transmembrane protein expressed in various tissues, including brown/beige adipocytes. Its tissue expression pattern is similar to that of UCP1 (a key marker of thermogenic adipocytes), suggesting a key role in regulating adipose tissue metabolism and function (38, 39). Specifically, *TMEM26* may modulate brown adipocyte function and enhance energy expenditure, thereby regulating body weight and lipid metabolism (40–42). Interestingly, *LRP1B* influences lipid metabolism through mechanisms involving rs431809 and CpG methylation (43). Additionally, it may promote lipogenesis via the AMPK signaling pathway, contributing to the regulation of lipid metabolism (44, 45). Finally, two studies have found that the protein encoded by *UBE2E2* plays a critical role in insulin secretion (46, 47). Overexpression of this gene regulates insulin secretory function by promoting the ubiquitination of proinsulin, ultimately impacting lipid metabolism and energy balance (48). The remaining 13 genes (*FAM13C, ZNF37A, RHOBTB1, BICC1, HNRNPF, TAF5L, URB2, FXYD4, PHYHIPL, RHOU, PGBD5, LOC110256649*, and *LOC110256821)* have not been directly linked to the regulation of the fatty acid content in mammals. Their specific roles in porcine muscle fatty acid metabolism and mechanisms of action require further validation.

We further analyzed genotype–phenotype associations and candidate gene transcriptome patterns. For example, at the oleic acid-associated SNP at Chr14:64941441 (near *TMEM26*), pigs with the G/A genotype had significantly higher oleic acid contents than those of pigs with G/G or A/A (*P* < 0.001). Given the role of *TMEM26* role in regulating brown adipocyte function, the G/A genotype of *TMEM26* may increase energy expenditure and promote fatty acid oxidation to regulate oleic acid accumulation in pork. In addition, the transcript levels of *ABCB10, RHOBTB1*, and *UBE2E2* in porcine tissues suggest that they contribute to fatty acid metabolism through distinct pathways. Specifically, *ABCB10* is highly expressed in the intestine, suggesting that it regulates intestinal fatty acid absorption to influence muscle lipid deposition. *RHOBTB1* is expressed in skeletal muscle, directly influencing fatty acid storage and metabolism in pigs (52, 53). *UBE2E2* is expressed in the hypothalamus, and its hypothalamic expression may regulate insulin secretion, thereby indirectly influencing lipid metabolism.

For future research, these SNPs and candidate genes identified through GWAS will require further functional validation. Specifically, we can employ gene editing techniques to perform knockout or overexpression of these candidate genes in adipocytes. Additionally, future studies should collect more samples, integrate transcriptomic and metabolomic data to decipher the regulatory mechanisms underlying fatty acid metabolism, and ultimately validate these markers in breeding programs to facilitate their application in pig genetic improvement.

## 5 Conclusions

In phenotypic analyses of three crossbred pig populations, the YT and YN populations had significantly higher TAA than that of the DT population (*P* < 0.05), while the DT population tended to exhibit significantly higher TFA, SFA, and UFA contents than those of the YT and YN populations (*P* < 0.05). A GWAS revealed 19 candidate genes associated with fatty acid metabolism, including six candidate genes (*ABCB10, GALNT2, RET, TMEM26, LRP1B*, and *UBE2E2*) and 13 newly identified genes (*ZNF37A, RHOBTB1, BICC1, HNRNPF, TAF5L, URB2, FXYD4, PHYHIPL, RHOU, PGBD5, LOC110256649*, and *LOC110256821*). The significant SNPs exhibited notable effects on fatty acid contents by genotype. Finally, transcriptional analyses and functional annotation clearly showed that different genes may collectively affect the fatty acid content through distinct pathways. These findings deepen our understanding of the genetic basis of fatty acid compositions in crossbred pigs and provide a basis for designing breeding schemes to genetically improve pork quality.

## Data Availability

The raw Whole Genome Sequencing data of ear tissues of YN pigs generated in this study are available in Sequence Read Archive (SRA) under BioProject PRJNA1303999. The raw Whole Genome Sequencing data of ear tissues of DT pigs generated in this study are available in Sequence Read Archive (SRA) under BioProject PRJNA1304141. The raw Whole Genome Sequencing data of ear tissues of YT pigs generated in this study are available in Sequence Read Archive (SRA) under BioProject PRJNA1303980.
